# Comparison of retinal nerve fiber layer thickness and Bruch’s membrane opening minimum rim width thinning rate in open-angle glaucoma

**DOI:** 10.1038/s41598-022-20423-0

**Published:** 2022-09-27

**Authors:** Donghee Park, Sung Pyo Park, Kyeong Ik Na

**Affiliations:** Department of Ophthalmology, Kangdong Sacred Heart Hospital, 150, Seongan-Ro, Gangdong-Gu, Seoul, 05355 South Korea

**Keywords:** Diseases, Medical research

## Abstract

This study aimed to compare the rate of thinning between retinal nerve fiber layer thickness (RNFLT) and Bruch’s membrane opening minimum rim width (BMO-MRW) in open-angle glaucoma (OAG) according to glaucoma severity. We retrospectively reviewed subjects with a total of 111 eyes with OAG that had undergone optical coherence tomography more than four times during more than 3 years of follow-up. The subjects were divided into three groups based on the mean deviation (MD) of the baseline visual field test: high MD (MD > − 2 dB), medium MD (− 2 dB ≥ MD > − 6 dB), and low MD (− 6 dB ≥ MD > − 12 dB) groups. A linear mixed model was employed to compare the rate of thinning between RNFLT and BMO-MRW among the three groups. The rate of RNFLT thinning was fastest in the inferotemporal sector in all three groups. The rate of BMO-MRW thinning was fastest in the inferotemporal sector of the high MD group and the superotemporal sector of the other two groups. Among the three groups, the rate of RNFLT thinning was not significantly different in the global sector and all sectors except the nasal sector. The rate of BMO-MRW thinning in the inferotemporal sector showed no significant difference, but that in the superotemporal sector was faster in the medium MD and low MD groups than in the high MD group. The fastest rate of RNFLT thinning was consistently observed in the inferotemporal sector, but BMO-MRW showed a change in the fastest thinning sector from inferotemporal to superotemporal, with increasing severity in early to moderate OAG. The difference in the changes in the two parameters may help understand the pathogenesis of glaucoma and predict its progression.

## Introduction

Glaucoma is a progressive optic neuropathy that causes morphological changes in the optic nerve head (ONH) and retinal nerve fiber layer (RNFL), which can result in irreversible visual field defects^1^. Longitudinally measured structural ONH changes or RNFL thickness (RNFLT) thinning are predictive of subsequent visual field deterioration. Optimal glaucoma management can be achieved by detecting early changes and long-term monitoring of the progression rate^2–5^.

Recently, it was reported that Bruch’s membrane opening minimum rim width (BMO-MRW), a reliable ONH parameter, would be more suitable for early detection of glaucomatous progression than RNFLT in eyes with disc hemorrhages^6^. In addition, a previous study showed that RNFLT was preferable for monitoring glaucomatous changes, whereas BMO-MRW was more sensitive for early detection of glaucoma damage^7^. A study reported that Bruch’s membrane opening minimum rim area (BMO-MRA), another ONH parameter, decreased as glaucoma progressed, which was followed by RNFLT thinning and a decrease of visual field index, in order^8^.

Based on the above studies, it can be assumed that when glaucoma progresses, the change in ONH morphology precedes RNFLT thinning, which makes it important to carefully monitor ONH changes in the early stages of glaucoma. However, to date, studies on the relationship between RNFLT and BMO-MRW changes are insufficient. In this study, we investigated the difference in the rate of thinning between RNFLT and BMO-MRW in open-angle glaucoma (OAG), according to the severity of glaucoma.

## Results

### Baseline characteristics

In total, data on 111 eyes of subjects with OAG were included in the study. The mean age of subjects at baseline examination was 59.01 ± 13.96 years and 61 (55.0%) were men. Of the total OAG, 98 (88.3%) eyes were diagnosed with normal-tension glaucoma (NTG), and the others were with primary open-angle glaucoma (POAG). The subjects completed 5.44 ± 1.58 optical coherence tomography (OCT) examinations during a mean follow-up duration of 3.54 ± 0.57 years. The mean spherical equivalent of all subjects was − 0.87 ± 2.77 diopters. Intraocular pressure (IOP) at baseline was 12.19 ± 3.17 mmHg and the mean follow-up IOP was 12.23 ± 2.63 mmHg with 1.70 ± 0.74 mmHg of fluctuation. The average axial length (AL) and central corneal thickness (CCT) were 24.19 ± 1.27 mm and 522.58 ± 36.79 µm, respectively. Baseline mean deviation (MD) was − 4.22 ± 3.85 dB and pattern standard deviation was 4.58 ± 3.30 dB (Table 1).

**Table 1:** Demographic and clinical characteristics of subjects with open-angle glaucoma.

Variables	MD > − 2 dB (H)	− 2 dB ≥ MD > − 6 dB (M)	− 6 dB ≥ MD > − 12 dB (L)	P-value	Post hoc	Total
Subjects, n (%)	35 (31.5)	48 (43.2)	28 (25.2)			111
Age at baseline examination, y	57.06 ± 12.18	56.52 ± 14.09	65.43 ± 13.90	0.015*	H, M < L	59.01 ± 13.96
Male, n (%)	19 (54.3)	25 (52.1)	12 (42.9)			61 (55.0)
**Systemic factors**						
Diabetes mellitus, n (%)	7 (20.0)	13 (27.1)	7 (25.0)	0.057*		27 (24.3)
Hypertension, n (%)	15 (42.9)	19 (39.6)	8 (28.6)	0.158*		42 (37.8)
**Glaucoma**						
Normal tension glaucoma, n (%)	32 (91.4)	39 (81.3)	27 (96.4)			98 (88.3)
Primary open-angle glaucoma, n (%)	3 (8.6)	9 (18.7)	1 (3.6)			13 (11.7)
Follow-up duration, y	3.76 ± 0.39	3.51 ± 0.59	3.33 ± 0.67	0.009*	H > M > L	3.54 ± 0.57
Follow-up OCT, n	5.20 ± 0.96	5.63 ± 1.91	5.43 ± 1.60	0.485*		5.44 ± 1.58
Spherical equivalent (diopter)	− 0.52 ± 2.95	− 1.29 ± 0.45	− 1.26 ± 0.48	0.443*		− 0.87 ± 2.77
IOP (mmHg)	12.57 ± 0.47	13.38 ± 0.51	11.79 ± 0.58	0.109*		12.19 ± 3.17
Mean follow-up IOP (mmHg)	11.89 ± 0.44	12.79 ± 11.98	11.36 ± 10.43	0.061*		12.23 ± 2.63
IOP fluctuation (mmHg)	1.61 ± 0.13	1.74 ± 0.12	1.63 ± 0.12	0.694*		1.70 ± 0.74
Peak follow-up IOP (mmHg)	13.77 ± 0.57	15.13 ± 0.50	13.54 ± 0.53	0.070*		14.48 ± 3.30
Axial length (mm)	24.58 ± 0.27	24.10 ± 0.21	24.32 ± 0.36	0.382*		24.19 ± 1.27
Central corneal thickness (µm)	521.63 ± 6.54	524.64 ± 5.38	522.65 ± 8.23	0.940*		522.58 ± 36.79
BMO area (mm^2^)	2.32 ± 0.08	2.16 ± 0.06	2.25 ± 0.08	0.290*		2.24 ± 0.43
Optic disc hemorrhage, n (%)	6 (17.1)	7 (14.6)	6 (21.4)	0.855*		19 (17.1)
**Baseline visual field examination**						
Mean deviation, dB	− 0.24 ± 0.93	− 3.81 ± 1.03	− 9.68 ± 2.66	< 0.001*	H > M > L	− 4.22 ± 3.85
Pattern standard deviation, dB	2.10 ± 0.88	4.15 ± 2.43	8.45 ± 3.49	< 0.001*	H < M < L	4.58 ± 3.30

MD, mean deviation; OCT, optical coherence tomography; IOP, intraocular pressure; BMO, Bruch’s membrane opening.

*One-way analysis of variance.

Of the 111 eyes of the subjects, 35 (31.5%), 48 (43.2%), and 28 (25.2%) were grouped into the high, medium, and low MD groups, respectively. The subjects in the low MD group were older comparatively. The higher MD group showed longer mean follow-up duration. Other demographic and clinical characteristics, except for visual field parameters, showed no significant differences, as shown in Table 1.

### Comparison of baseline RNFLT and BMO-MRW

The baseline global and six Garway-Heath sectoral RNFLT and BMO-MRW showed significant differences among the three groups (all *p* < 0.001, except nasal BMO-MRW for *p* = 0.002). The RNFLT decreased with increasing glaucoma severity, except in the nasal sector, where there was no significant difference between the medium and low MD groups. BMO-MRW decreased as severity increased in the global and three Garway-Heath sectors (superotemporal, inferotemporal, and inferonasal). There was no significant difference in BMO-MRW between the high and medium MD groups in the temporal and superonasal sectors and between the medium and low MD groups in the nasal sector (Table 2).

**Table 2:** Comparison of baseline retinal nerve fiber layer thickness and Bruch’s membrane opening minimum rim width of subjects with open-angle glaucoma grouped by mean deviation.

Variables	MD > − 2 dB (H)	− 2 dB ≥ MD > − 6 dB (M)	− 6 dB ≥ MD > − 12 dB (L)	P-value	Post hoc
**Baseline RNFLT characteristics (µm)**					
Average global RNFLT	90.36 ± 10.01	80.04 ± 15.61	72.44 ± 13.74	< 0.001*	H > M > L
Average superotemporal RNFLT	115.71 ± 26.80	94.20 ± 28.76	79.46 ± 29.09	< 0.001*	H > M > L
Average temporal RNFLT	71.25 ± 13.14	66.00 ± 16.15	59.36 ± 17.09	< 0.001*	H > M > L
Average inferotemporal RNFLT	134.20 ± 25.63	111.22 ± 35.16	89.81 ± 39.28	< 0.001*	H > M > L
Average inferonasal RNFLT	99.51 ± 20.18	87.59 ± 23.91	79.52 ± 25.43	< 0.001*	H > M > L
Average nasal RNFLT	72.29 ± 12.64	65.93 ± 15.26	64.70 ± 11.88	< 0.001*	H > M, L
Average superonasal RNFLT	104.20 ± 22.63	97.84 ± 31.10	91.97 ± 25.31	< 0.001*	H > M > L
**Baseline BMO-MRW characteristics (µm)**					
Average global BMO-MRW	211.55 ± 39.86	199.63 ± 46.41	181.97 ± 35.18	< 0.001*	H > M > L
Average superotemporal BMO-MRW	208.98 ± 44.71	186.78 ± 60.64	168.14 ± 52.37	< 0.001*	H > M > L
Average temporal BMO-MRW	148.89 ± 31.89	152.96 ± 42.96	130.95 ± 35.43	< 0.001*	H, M > L
Average inferotemporal BMO-MRW	236.92 ± 52.22	209.89 ± 61.91	176.53 ± 62.81	< 0.001*	H > M > L
Average inferonasal BMO-MRW	261.66 ± 60.97	239.42 ± 58.70	218.43 ± 66.29	< 0.001*	H > M > L
Average nasal BMO-MRW	226.74 ± 53.66	213.26 ± 56.80	205.55 ± 54.25	0.002*	H > M, L
Average superonasal BMO-MRW	239.48 ± 46.24	229.72 ± 62.68	214.75 ± 44.81	< 0.001*	H, M > L

MD, mean deviation; RNFLT, retinal nerve fiber layer thickness; BMO-MRW, Bruch’s membrane opening minimum rim width.

*One-way analysis of variance.

### Comparison of the rate of thinning of RNFLT and BMO-MRW

RNFLT and BMO-MRW decreased significantly in the global and six Garway-Heath sectors (all *p* < 0.001). The fastest rate of thinning per year was − 2.29 ± 0.43 µm in the inferotemporal sector of RNFLT and − 3.88 ± 0.42 µm in the superotemporal sector of BMO-MRW, respectively (Table 3).

**Table 3:** Rate of thinning of retinal nerve fiber layer thickness and Bruch’s membrane opening minimum rim width of all subjects with open-angle glaucoma in global and six Garway-Heath sectors.

Variables	Total	P-value
**Rate of RNFLT thinning (µm/y)**		
Rate of global RNFLT thinning	− 1.19 ± 0.09	< 0.001^†^
Rate of superotemporal RNFLT thinning	− 1.92 ± 0.17	< 0.001^†^
Rate of temporal RNFLT thinning	− 0.96 ± 0.10	< 0.001^†^
Rate of inferotemporal RNFLT thinning	− 2.29 ± 0.43*	< 0.001^†^
Rate of inferonasal RNFLT thinning	− 1.09 ± 0.16	< 0.001^†^
Rate of nasal RNFLT thinning	− 0.76 ± 0.12	< 0.001^†^
Rate of superonasal RNFLT thinning	− 1.29 ± 0.16	< 0.001^†^
**Rate of BMO-MRW thinning (µm/y)**		
Rate of global BMO-MRW thinning	− 2.59 ± 0.24	< 0.001^†^
Rate of superotemporal BMO-MRW thinning	− 3.88 ± 0.42*	< 0.001^†^
Rate of temporal BMO-MRW thinning	− 2.32 ± 0.25	< 0.001^†^
Rate of inferotemporal BMO-MRW thinning	− 3.78 ± 0.38	< 0.001^†^
Rate of inferonasal BMO-MRW thinning	− 2.72 ± 0.33	< 0.001^†^
Rate of nasal BMO-MRW thinning	− 2.10 ± 0.32	< 0.001^†^
Rate of superonasal BMO-MRW thinning	− 2.70 ± 0.46	< 0.001^†^

RNFLT, retinal nerve fiber layer thickness; BMO-MRW, Bruch’s membrane opening minimum rim width.

*The highest value among the global and six Garway-Heath sectors.

^†^Linear mixed model.

**Figure 1:**
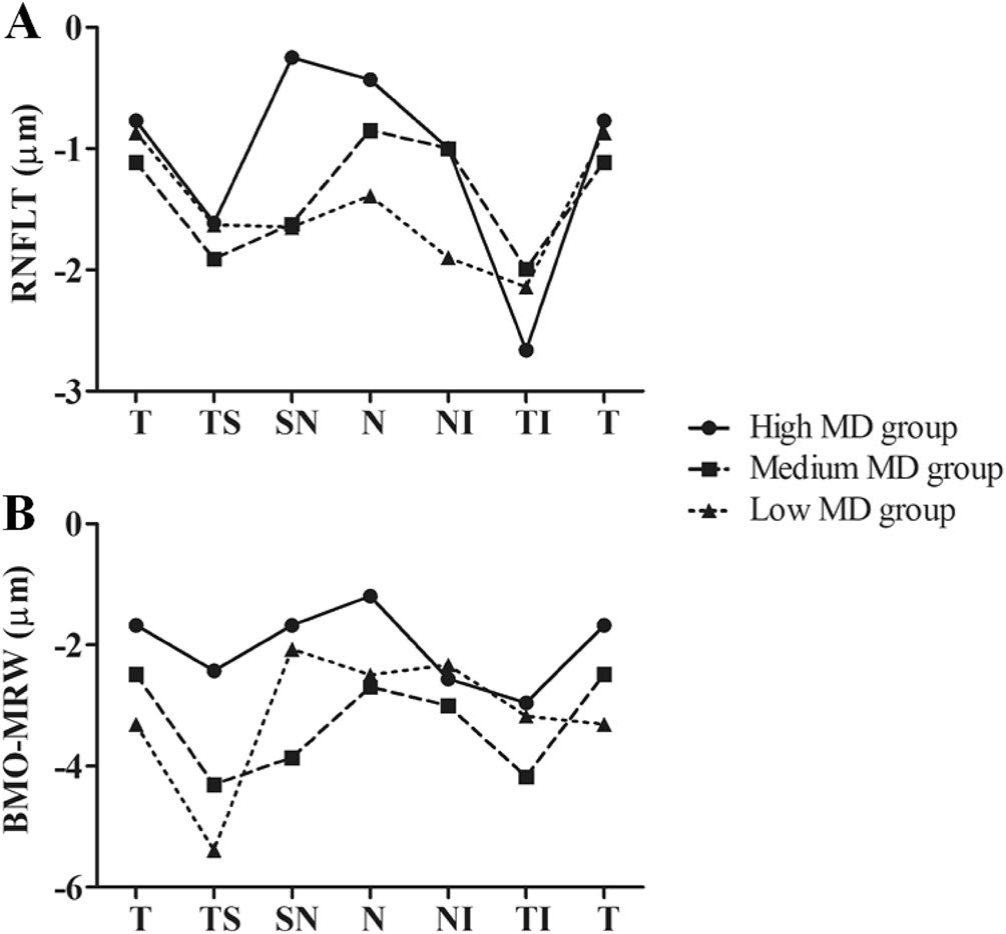
Comparison of the rate of thinning of (**A**) retinal nerve fiber layer thickness and (**B**) Bruch’s membrane opening minimum rim width in each Garway-Heath sectors among the three groups: high mean deviation (MD > − 2 dB), medium MD (− 2 dB ≥ MD > − 6 dB), and low MD (− 6 dB ≥ MD > − 12 dB). RNFLT, retinal nerve fiber layer thickness; BMO-MRW, Bruch’s membrane opening minimum rim width; MD, mean deviation; T, temporal; TS, superotemporal; SN, superonasal; N, nasal; NI, inferonasal; TI, inferotemporal.

**Figure 2:**
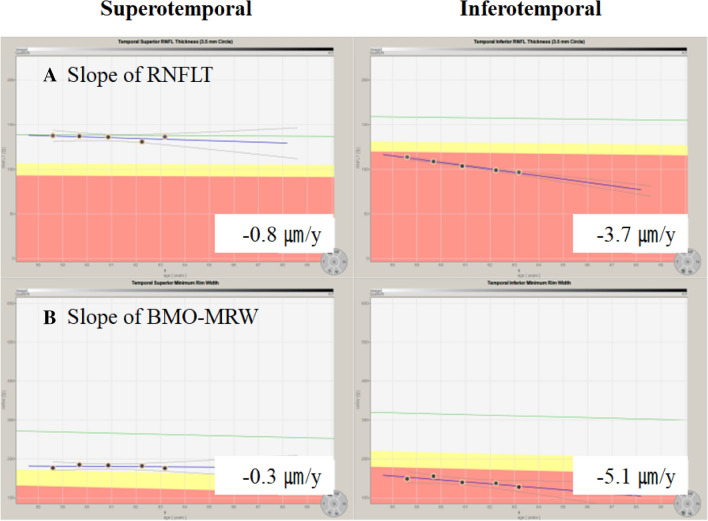
Representative case showing the rate of thinning of retinal nerve fiber layer thickness (RNFLT) and Bruch’s membrane opening minimum rim width (BMO-MRW) in the superotemporal and inferotemporal sectors of a 58-year-old woman with a baseline mean deviation of − 0.53 dB from March 2017 to November 2020. The slope of RNFLT (**A**), − 3.7 µm/y, is faster in the inferotemporal sector than that of − 0.8 µm/y in the superotemporal sector. In addition, the slope of BMO-MRW (**B**), − 5.1 µm/y, is faster in the inferotemporal sector than that of − 0.3 µm/y in the superotemporal sector. RNFLT, retinal nerve fiber layer thickness; BMO-MRW, Bruch’s membrane opening minimum rim width.

**Figure 3:**
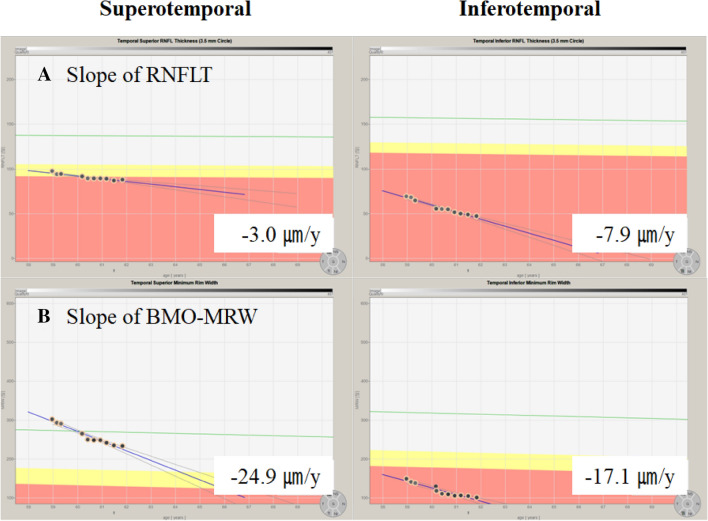
Representative case showing the rate of thinning of retinal nerve fiber layer thickness (RNFLT) and Bruch’s membrane opening minimum rim width (BMO-MRW) in the superotemporal and inferotemporal sectors of a 57-year-old woman with a baseline mean deviation of − 8.63 dB from December 2017 to January 2021. The slope of RNFLT (**A**), − 7.9 µm/y, is faster in the inferotemporal sector than of − 3.0 µm/y in the superotemporal sector. In addition, the slope of BMO-MRW (**B**), − 24.9 µm/y, is faster in the superotemporal sector than that of -17.1 µm/y in the inferotemporal sector. RNFLT, retinal nerve fiber layer thickness; BMO-MRW, Bruch’s membrane opening minimum rim width.

In the inner-group analysis classified by MD values, RNFLT and BMO-MRW showed significant thinning in the global and most sectors (all *p* < 0.05, except superonasal RNFLT in the high MD group for *p* = 0.693 and superonasal BMO-MRW in the low MD group for *p* = 0.065). The rate of RNFLT thinning was fastest in the inferotemporal sector in all three groups (− 2.66 µm/y, *p* = 0.022; − 1.99 µm/y, *p* < 0.001; and − 2.14 µm/y, *p* < 0.001 in high, medium, and low MD groups, respectively) (Fig. 1A). On the other hand, BMO-MRW thinning was fastest in the inferotemporal sector of the high MD group (− 2.95 µm/y, *p* < 0.001), and in the superotemporal sector of medium and low MD groups (− 4.30 µm/y, *p* < 0.001; − 5.39 µm/y, *p* < 0.001, respectively) (Fig. 1B, Table 4). Figure 2 illustrates RNFLT and BMO-MRW changes in the superotemporal and inferotemporal sectors of a 58-year-old woman in the high MD group with a baseline MD of − 0.53 dB, while Fig. 3 illustrates a representative case of the low MD group showing a 57-year-old woman with the baseline MD of − 8.63 dB.

**Table 4:** Comparison of the rate of thinning of retinal nerve fiber layer thickness and Bruch’s membrane opening minimum rim width of subjects with open-angle glaucoma grouped by mean deviation.

Variables	MD > − 2 dB (H)	− 2 dB ≥ MD > − 6 dB (M)	− 6 dB ≥ MD > − 12 dB (L)	P-value		
**Rate of RNFLT thinning**^**†**^** (µm/y)**						
Rate of global RNFLT thinning	− 0.94 (p < 0.001)	− 1.26 (p < 0.001)	− 1.71 (p = 0.005)	0.192^‡^	0.073^§^	0.458^¶^
Rate of superotemporal RNFLT thinning	− 1.61 (p = 0.021)	− 1.91 (p < 0.001)	− 1.63 (p < 0.001)	0.338^‡^	0.412^§^	0.507^¶^
Rate of temporal RNFLT thinning	− 0.77 (p = 0.003)	− 1.11 (p < 0.001)	− 0.87 (p = 0.001)	0.329^‡^	0.788^§^	0.279^¶^
Rate of inferotemporal RNFLT thinning	− 2.66 (p = 0.022)*	− 1.99 (p < 0.001)*	− 2.14 (p < 0.001)*	0.511^‡^	0.864^§^	0.806^¶^
Rate of inferonasal RNFLT thinning	− 1.00 (p = 0.040)	− 1.00 (p < 0.001)	− 1.90 (p = 0.004)	0.982^‡^	0.094^§^	0.130^¶^
Rate of nasal RNFLT thinning	− 0.43 (p = 0.014)	− 0.85 (p < 0.001)	− 1.39 (p = 0.033)	0.012^‡^	0.040^§^	0.138^¶^
Rate of superonasal RNFLT thinning	− 0.25 (p = 0.693)	− 1.62 (p < 0.001)	− 1.65 (p < 0.001)	0.119^‡^	0.077^§^	0.910^¶^
**Rate of BMO-MRW thinning**^**†**^** (µm/y)**						
Rate of global BMO-MRW thinning	− 1.62 (p < 0.001)	− 3.24 (p < 0.001)	− 2.94 (p < 0.001)	0.003^‡^	0.073^§^	0.706^¶^
Rate of superotemporal BMO-MRW thinning	− 2.42 (p < 0.001)	− 4.30 (p < 0.001)*	− 5.39 (p < 0.001)*	0.027^‡^	0.006^§^	0.284^¶^
Rate of temporal BMO-MRW thinning	− 1.67 (p < 0.001)	− 2.48 (p < 0.001)	− 3.31 (p < 0.001)	0.208^‡^	0.013^§^	0.330^¶^
Rate of inferotemporal BMO-MRW thinning	− 2.95 (p < 0.001)*	− 4.17 (p < 0.001)	− 3.17 (p = 0.001)	0.155^‡^	0.894^§^	0.184^¶^
Rate of inferonasal BMO-MRW thinning	− 2.56 (p < 0.001)	− 3.00 (p < 0.001)	− 2.33 (p = 0.005)	0.517^‡^	0.746^§^	0.354^¶^
Rate of nasal BMO-MRW thinning	− 1.19 (p = 0.024)	− 2.69 (p < 0.001)	− 2.49 (p = 0.002)	0.040^‡^	0.156^§^	0.763^¶^
Rate of superonasal BMO-MRW thinning	− 1.67 (p = 0.047)	− 3.86 (p < 0.001)	− 2.07 (p = 0.065)	0.045^‡^	0.799^§^	0.144^¶^

MD, mean deviation; RNFLT, retinal nerve fiber layer thickness; BMO-MRW, Bruch’s membrane opening minimum rim width.

*The highest value among the global and six Garway-Heath sectors.

^†^Linear mixed model.

^‡^Linear mixed model, mean deviation > − 2 dB versus − 2 dB ≥ mean deviation > − 6 dB (M).

^§^Linear mixed model, mean deviation > − 2 dB versus − 6 dB ≥ mean deviation > − 12 dB.

^¶^Linear mixed model, − 2 dB ≥ mean deviation > − 6 dB (M) versus − 6 dB ≥ mean deviation > − 12 dB.

**Figure 4:**
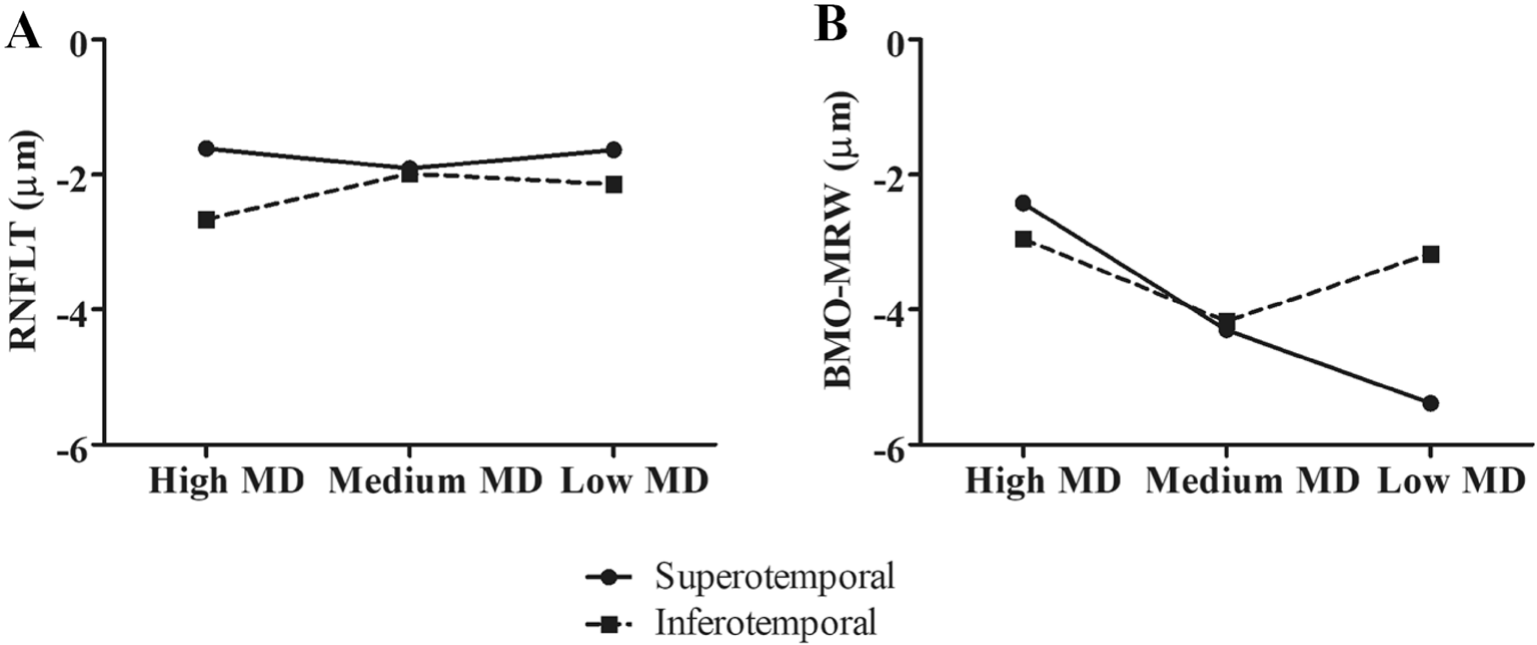
Comparison of the rate of thinning of (**A**) retinal nerve fiber layer thickness and (**B**) Bruch’s membrane opening minimum rim width of high mean deviation (MD > − 2 dB), medium MD (− 2 dB ≥ MD > − 6 dB), and low MD (− 6 dB ≥ MD > − 12 dB) groups in the superotemporal and inferotemporal sectors. RNFLT, retinal nerve fiber layer thickness; BMO-MRW, Bruch’s membrane opening minimum rim width; MD, mean deviation.

In the inter-group analysis, RNFLT did not show a significant difference between the groups in global and all Garway-Heath sectors, except the nasal sector. In BMO-MRW, there was no difference in the rate of thinning in the inferotemporal sector. Whereas the superotemporal sector showed significant faster rate of BMO-MRW thinning as glaucoma progressed; − 2.42 µm/y in the high MD group, − 4.30 µm/y in the medium MD group, and − 5.39 µm/y in the low MD group (*p* = 0.027 for high MD group vs medium MD group; *p* = 0.006 for high MD group vs low MD group) (Fig. 4, Table 4).

## Discussion

To our knowledge, the present study is the first to investigate the difference in the rate of thinning between RNFLT and BMO-MRW in OAG according to glaucoma severity. In early to moderate OAG with MD > − 12 dB, the fastest rate of RNFLT thinning was constant in the inferotemporal sector; however, in BMO-MRW, it moved from the inferotemporal to the superotemporal sector as glaucoma severity increased.

Although optic disc stereophotographs have been considered as gold standard for the evaluation of structural damage in glaucoma, they showed limitation in qualitative and subjective properties for evaluating the progression rate. Therefore, advances have been made in imaging technologies to detect and quantify glaucomatous progression. In particular, OCT provides objective and repeatable measurements related to ONH and RNFLT^9^.

Previous studies have compared the rate of thinning between RNFLT and BMO-MRW with serial OCT examinations^6,10^. Cho and Kee^6^ compared the progression rates of RNFLT and BMO-MRW in eyes with disc hemorrhage. They found that the rate of thinning of BMO-MRW was faster than that of RNFLT, especially in the inferotemporal and superotemporal sectors, and BMO-MRW would be more suitable for detecting glaucoma progression than RNFLT^6^. They also reported in another study that BMO-MRW showed a more noticeable reduction rate than RNFLT, which resulted in greater diagnostic power in early normal tension glaucoma with MD > − 6 dB in which the visual field may not show significant change^10^. The above studies analyzed the relationship between the changes in RNFLT and BMO-MRW in eyes with disc hemorrhage and early glaucoma. In the present study, we classified subjects according to glaucoma severity using MD and compared the progression rates. Kostanyan et al.^11^ reported that baseline glaucoma severity had a significant impact on glaucoma progression rate. In addition, Medeiros et al.^12^ showed that changes in retinal ganglion cell counts resulted in different effects on RNFLT and MD, depending on the severity of glaucoma. Therefore, disease severity should be considered when analyzing the progression rate of glaucoma.

The progression of RNFLT and BMO-MRW has a temporal relation; a significant thinning of BMO-MRW is followed by that of RNFLT^13–15^. In a non-human primate study, the average surface height of the ONH changed prior to reduction of global RNFLT^16^. Choi et al.^8^ reported that as OAG progressed, the first structural change was observed in BMO-MRA, which was followed by RNFLT and visual field, in order. The response of the ONH to IOP changes could explain the temporal relationship between RNFLT and BMO-MRW. Sharma et al.^17^ found that BMO-MRW in patients with glaucoma would decrease with elevated IOP, and acute IOP increase led to compression of the neuroretinal rim without affecting the ONH size. This implies that the ONH structure was first under mechanical stress, followed by RNFL damage in glaucoma. Therefore, as glaucoma progressed, ONH or BMO-MRW change would precede RNFLT thinning.

Leung et al.^18^ reported that RNFL defects in glaucoma were most frequently observed in the inferotemporal sector, followed by the superotemporal sector. Kim et al.^19^ analyzed early to moderate OAG with more than MD − 12 dB and showed that significantly higher rates of thinning were observed on average at 6 and 11 o’clock hours of RNFLT, especially with the highest rate at 6 o’clock in subjects showing structural and functional progression. Furthermore, Hood et al.^20^ classified glaucoma subjects into three groups based on MD − 1.5 dB and − 5.5 dB and found that the thinning of RNFL and retinal ganglion cell plus inner plexiform layer was significantly greater in the inferior region compared to the superior, which could be attributed to the structural difference between the optic nerve and the macula.

In this study, we aimed to evaluate the difference in the change in the thinning rate between RNFLT and BMO-MRW according to glaucoma severity. We found that there was a difference between the two parameters in the sector that showed the fastest rate of thinning as glaucoma progressed. As mentioned above, BMO-MRW showed structural changes prior to RNFLT as glaucoma progressed, and the changes generally occurred first in the inferotemporal sector, followed by the superotemporal sector. According to our findings, RNFLT continued to show the fastest thinning in the inferotemporal sector, despite glaucoma progression. However, BMO-MRW showed a change in the fastest thinning sector from inferotemporal to superotemporal, which could be considered to be preceding the RNFLT.

A limitation of this study is that it was based on a single ethnicity of Koreans; therefore, it may be difficult to generalize the results to other ethnicities. There was a difference in race-related rates of thinning of global BMO-MRW, and race should be considered when comparing glaucoma progression^11,21^. Further research including other ethnicities is needed in the future.

In conclusion, we found that the fastest rate of RNFLT thinning was constant in the inferotemporal sector of RNFLT, but that of BMO-MRW was shifted from the inferotemporal to the superotemporal sector as the severity of glaucoma increased in OAG with MD > − 12 dB. The difference between the two parameters over time may help to understand the pathogenesis of glaucoma and predict its progression.

## Methods

This retrospective study adhered to the tenets of the Declaration of Helsinki and was approved by the Institutional Review Board (IRB) of Kangdong Sacred Heart Hospital. Informed consent was waived for the study due to its retrospective nature, which was confirmed by IRB of Kangdong Sacred Heart Hospital (IRB No. 2022-04-007).

### Subjects

The present study retrospectively reviewed the medical records of patients with OAG who visited the Department of Ophthalmology at Kangdong Sacred Heart Hospital between March 2017 and February 2021.

Subjects satisfied the inclusion criteria when they had been followed up for over 3 years and completed thorough ophthalmic evaluation including more than four instances of OCT (Spectralis OCT; Heidelberg Engineering Inc., Heidelberg, Germany).

At baseline, all subjects underwent history taking and ophthalmic examinations, including best-corrected visual acuity, automated refraction, Goldmann applanation tonometry, slit-lamp biomicroscopy, gonioscopy, and measurement of the AL and CCT (OA-2000 optical biometer; Tomey, Nagoya, Japan), and standard automated perimetry (SAP) (Humphrey Field Analyzer, HFA II; Car Zeiss Meditec, Inc., Dublin, CA, USA) using the 24-2 Swedish Interactive Threshold Algorithm standard strategy.

OAG was defined as an open angle on gonioscopy and the presence of glaucomatous optic neuropathy on slit-lamp biomicroscopy, color fundus photographs, red-free fundus photographs, and corresponding glaucomatous visual field defects. Glaucomatous optic neuropathy was considered to be a focal neuroretinal rim thinning or notching, or generalized loss of the neuroretinal rim. Glaucomatous visual field defects were defined when at least two of the following three criteria were satisfied in more than one reliable visual field test (false-positive error of < 15%, false-negative error of < 15%, and fixation loss of < 20%): (1) a cluster of three points with < 5% probability on the pattern deviation map in at least one hemifield, with at least one point with a probability of < 1%; (2) a glaucoma hemifield test result outside normal limits; and (3) a pattern standard deviation outside 95% of the normal limits. NTG was defined as an OAG with an untreated baseline IOP ≤ 21 mmHg. POAG was defined as an OAG with an untreated baseline IOP > 21 mmHg.

The exclusion criteria were as follows: presence of any anterior segment conditions that may lower OCT image quality; a history of intraocular surgery except uncomplicated cataract surgery; any history of optic nerve diseases other than glaucoma; a history of systemic or neurologic diseases that may affect the optic disc or visual field; and any retinal diseases that led to retinal edema followed by swelling of the optic disc or RNFL. All OCT images were manually checked, and those with image quality scores less than 20 or segmentation errors were excluded.

The subjects were divided into three groups based on MD of SAP: ‘MD > − 2 dB’, ‘− 2 dB ≥ MD > − 6 dB’, ‘− 6 dB ≥ MD > − 12 dB’, which were named ‘high MD group’, ‘medium MD group’, and ‘low MD group’, respectively, in this study.

### Evaluation of RNFLT

RNFLT was measured using OCT with a 3.5 mm diameter peripapillary circle centered at the disc, and the thickness values were divided into the global sector and the following six sectors: superotemporal, temporal, inferotemporal, inferonasal, nasal, and superonasal.

### Evaluation of BMO-MRW

For the measurement of BMO-MRW using OCT, the Bruch’s membrane opening (BMO) plane was delineated in twenty-four radial B-scans, each covering a 15° region centered on the ONH; with two for each of the twenty-four delineated B-scans, a total of forty-eight rim width measurements were obtained. BMO-MRW was defined as the closest distance from the delineated BMO point to the inner limiting membrane within each radial B-scan, and a total forty-eight BMO-MRW were measured. BMO-MRW of the global and six Garway-Heath sectors was calculated.

### Statistical analysis

ANOVA was used to compare baseline demographics, clinical characteristics, and baseline RNFLT and BMO-MRW among the three groups.

The rates of RNFLT and BMO-MRW thinning in the global and Garway-Heath sectors were analyzed using the linear mixed-effects model. Models were adjusted with fixed coefficients (fixed effects) of age, sex, systemic factors (diabetes mellitus and hypertension), type of glaucoma (NTG and POAG), spherical equivalent, IOP, mean follow-up IOP, IOP fluctuation (standard deviation of IOP during the follow-up period), peak follow-up IOP, CCT, AL, BMO area, and optic disc hemorrhage, accepting random coefficients.

*P* values of < 0.05 were considered statistically significant. IBM SPSS ver. 21.0 (IBM Corp., Armonk, NY, USA) was used for all statistical analyses.

## Data Availability

The datasets generated during the current study are available from the corresponding author upon request.
